# Acute psychosis and serotonin syndrome in the setting of “Triple-C” overdose: a case report

**DOI:** 10.1186/s13256-021-03163-z

**Published:** 2021-11-04

**Authors:** Roudi Bachar, John Robert Majewski, Christopher Shrack, Anthony El-Khoury

**Affiliations:** Indiana University School of Medicine, 520 S. St, Vincennes, Indiana 47591 USA

**Keywords:** Overdose, Dextromethorphan hydrobromide, Serotonin syndrome, Psychosis

## Abstract

**Background:**

Over-the-counter medication overdose is a difficult diagnostic challenge for many physicians as common drug screening assays cannot detect these substances. We present a case of acute psychosis, serotonin syndrome, and anticholinergic overdose-like properties in the setting of Coricidin HBP Cough & Cold tablets, known by their street name Triple-C. This is the first case report we are aware of involving a patient presenting with these symptoms and requiring critical-care-level support.

**Case presentation:**

A 31-year-old African American female with a past medical history of anxiety, childhood asthma, previous methamphetamine abuse, and coronavirus disease 2019 infection in August 2020 was brought to the emergency department by the local police department with altered mental status. Initial blood work, including extended drug screens, were unremarkable for a definitive diagnosis. This patient required critical-care-level support and high sedation because of her symptoms. Collateral history revealed the patient regularly consumed Triple-C daily for the 6 weeks prior to admission. A trial off sedation was attempted after 24 hours with no complications. The patient admitted to regular Triple-C consumption and auditory hallucinations since adolescence. She was discharged safely after 48 hours back into the community. She was lost to follow-up with psychiatry and internal medicine; however, she was evaluated in the emergency room 1 month later with a similar psychiatric presentation.

**Conclusion:**

Overdose of Triple-C should be kept in the differential diagnosis of patients presenting with a triad of psychosis, serotonin syndrome, and anticholinergic overdose, in the setting of unknown substance ingestion.

## Introduction

Dextromethorphan hydrobromide (DXM) is a popular antitussive medication in the USA. It is commonly combined with other medications, including chlorpheniramine maleate (CPM). Coricidin HBP Cough & Cold tablets, otherwise known by their street name Triple-C, is a common drug that is abused for its euphoric properties [1, 2]. Over-the-counter medication overdose can present as a diagnostic challenge in the acute setting. One of the most serious adverse effects of Triple-C is due to the anticholinergic properties of CPM [3] and serotonergic effects of DXM [4]. Acute overdose of Triple-C can present as an anticholinergic overdose, serotonin syndrome, or respiratory depression [4–6]. We report a patient who presented with acute psychosis, anticholinergic overdose, and serotonin syndrome in the setting of habitual Triple-C abuse who may have had an underlying undiagnosed psychiatric disorder.

## Case presentation

A 31-year-old African American female with a past medical history of anxiety, childhood asthma, previous methamphetamine abuse, and coronavirus disease 2019 (COVID-19) infection in August 2020 was brought to the emergency department (ED) by the local police department with altered mental status. The patient is a halfway-house resident who was reportedly sober for 90 days.

ED vital signs showed a heart rate of 166 beats per minute, blood pressure 125/94 mmHg, respiratory rate 24 breaths per minute, 90% oxygen saturation on room air, and temperature 103.5 °F. Physical examination and full history were unable to be performed due to her agitation. She required intramuscular ketamine twice in order to be sedated and the assistance of multiple police officers to restrain the patient for safety.

Blood work revealed white blood cell count (WBC) of 7.4/μL (normal range 4.0–10.8/μL), hemoglobin 13.50 g/dL (normal range 11.8–14.8 g/dL), and platelet count 440/μL (normal range 140–350/μL). Comprehensive metabolic panel (CMP) revealed potassium of 3.4 mmol/L (normal range 3.5–5.5 mmol/L), bicarbonate 16 mmol/L (normal range 20–31 mmol/L), creatinine 1.33 mg/dL (normal range 0.55–1.02 mg/dL), and glucose 213 mg/dL (normal range 74–106 mg/dL). Other lab values included ammonia of 204 μmol/L (normal range 19–54 μmol/L), lactic acid > 12.20 mmol/L (normal range 0.5–1.9 mmol/L), lipase 61 U/L (normal range 12–53 U/L), and creatinine kinase 206 U/L (normal range 34–145 U/L). Beta-human chorionic gonadotropin (β-hCG), troponins, procalcitonin, urine drug screen including phencyclidine (PCP), extended serum drug screen, cerebrospinal fluid, alcohol, and Tylenol levels were negative/within normal limits. Urinalysis showed moderate blood, glucose 70 mg/dL (normal range 0 mg/dL), and protein 300 mg/dL (normal range 0 mg/dL). Arterial blood gas (ABG) revealed pH 7.365 (normal range 7.35–7.45), pCO_2_ 39.6 mmHg (normal range 35–45 mmHg), pO_2_ 154 mmHg (normal range 80–100 mmHg), bicarbonate 22.1 mmHg (normal range 22–26 mmHg), and lactate 0.8 mmol/L (normal range 0.5–1.9 mmol/L). Computed tomography (CT) of the head without contrast and CT chest abdomen and pelvis were negative for acute abnormalities.

The patient required large amounts of sedatives because of her agitation. Multiple attempts were made to titrate sedating medication to normal mentation or decreased agitation. Unfortunately, the patient did not respond to increasing doses of sedative medication to the point of normal mentation and continued to be aggressive and violent towards staff. It was agreed upon by the emergency medicine physicians and critical care team that she would need to be sedated, intubated, and ventilated due to her extreme agitation and aggression. Rapid sequence intubation was uneventful, and the patient was moved from the emergency department to the intensive care unit. She was started on propofol and fentanyl infusions with intravenous maintenance fluids. The patient continued to be agitated while sedated and required a third agent in the form of midazolam for adequate sedation. Management was primarily supportive, including temperature regulation (hyperthermia), intubation and mechanical ventilation (agitation and tachypnea), intravenous fluids (hypotension), and sedation (agitation). Serial ABGs were unremarkable with no lactic acidosis.

Collateral history obtained from co-residents from the patient’s halfway house revealed that the patient was in possession of large amounts of the common over-the-counter cough medication Coricidin HBP Cough & Cold tablets. The recovery home did not report her having any cough or upper respiratory symptoms in the 4 weeks prior to admission. She was diagnosed with Triple-C intoxication and overdose, complicated by serotonin syndrome and acute psychosis.

After 24 hours of critical care support, a trial off sedation was attempted. A spontaneous awaken trial was performed. The patient awoke with normal mentation and appropriate behavior. After a successful spontaneous awakening trial, a spontaneous breath trial was performed. The patient was able to protect her airway, had appropriate inspiratory and expiratory effort, and was able to maintain SpO_2_ > 92%. She was successfully liberated from mechanical ventilation with no complications.

When asked about the Triple-C, she stated that she had been using approximately six tablets twice daily for the past few weeks. Upon further discussion, she reported that her acute psychosis episode started after hearing voices in her head. She stated these voices had been present since adolescence, although never aggressive in nature. Her agitation and harmful ideation were the first instance of its kind.

She was evaluated by psychiatry, who deemed her fit for discharge. She did not present to her scheduled clinic appointments and was lost to follow-up in the outpatient setting to both psychiatry and internal medicine.

## Discussion and conclusions

We found this to be an interesting case of intoxication with an over-the-counter substance. There are previous reports of DXM-CPM-induced psychotic episodes in patients with established psychiatric disorders [5]. It is unclear in the literature if Triple-C-induced psychosis occurs only in patients with underlying psychiatric disorders or if it can produce psychosis *de novo*. Further research is required to determine this relationship. When she was interviewed by the inpatient psychiatry team, she denied hearing voices at that time. It was determined that she may have an underlying psychiatric pathology but would need further evaluation upon discharge. Unfortunately, she was lost to follow-up in the clinic. She presented to the emergency department with an acute psychotic episode less than 1 month later.

The patient denied an acute increase in her Triple-C use. However, as the patient’s urine drug screen, including an extended drug screen that was sent to an outside facility for testing, was negative, we attribute her acute psychosis to the ingestion of large amounts of Triple-C. There is evidence in the literature that DXM-CMP metabolization can vary widely within individuals [7], which also may have contributed to her sudden symptomatology. At high doses, DXM has a similar pharmacological effect as PCP and produces similar behavioral manifestations [5]. It can even cause a false-positive on certain drug assays for PCP [8]. Serotonin syndrome is a disease of neuro-hyperexcitability of the 5-Ht subgroup receptors. Patients classically present with tachycardia and hypertension as well as hyperthermia, agitation, ocular clonus, dilated pupils, tremor, hyperreflexia, spontaneous muscle clonus, and dry mucus membranes [9]. Anticholinergic overdose is due to competitive antagonism of muscarinic receptors. These are primarily located in the central nervous system and are responsible for cognitive function and delirium. Peripheral muscarinic receptors are responsible for multiple functions in the autonomic nervous system [10]. This combination of central and peripheral receptor antagonism is the mechanism for the manifestations of agitation, delirium, hyperthermia, and visual perception abnormalities. The hyperthermia, tachycardia, tachypnea, hypertension, and altered mental status are most likely due to the synergistic anticholinergic and serotonergic properties of DXM-CMP.

This case is important as it provides another differential for clinicians to keep in mind in the acutely psychotic patient with both anticholinergic and serotonergic signs and symptoms in the setting of a negative drug screen. Patients may even require critical-care-level support. However, these drugs are metabolized relatively quickly, and trial off sedation can be attempted in less than 24 hours. The other learning point is the importance of a collateral history. This prevents the treatment team from administering costly and even harmful antidotes that will provide no therapeutic effect. The family of the patient, recovery home mentor, and recovery home administrators were all crucial in ruling in and out different etiologies. Multidisciplinary teams are not just limited to medical staff within a hospital system. Social staff outside of the immediate clinical setting can provide valuable information and often know the patient better than medical staff.

## Data Availability

Any supporting data can be obtained by direct correspondence with the corresponding author (RB).

## Abbreviations

ABG: Arterial blood gas
CPM: Chlorpheniramine maleate
CBC: Complete blood count
CMP: Comprehensive metabolic panel
Triple-C: Coricidin HBP Cough & Cold tablets
DXM: Dextromethorphan hydrobromide
PCP: Phencyclidine
